# Margarine Adulteration

**Published:** 1893-02-04

**Authors:** E. W. Hope

**Affiliations:** Assistant Med. Officer. Sanitary Department, Municipal Buildings, Dale Street, Liverpool


### EDITOR'S LETTER-BOX.

[Our correspondents are reminded that prolixity is a great bar to publication, and that brevity of style and conciseness of statement greatly facilitate early insertion.]

### MARGARINE ADULTERATION.

SIR,—My attention has been directed to a paragraph in your newspaper headed "Four Years of Margarine," in which you compare the number of prosecutions under the Margarine Act in Liverpool with the number elsewhere, and drew the conclusion that the excess of such prosecutions in Liverpool shows that "Liverpool has reached the bad eminence of being the worst city in the Kingdom in the margarine imposture." The facts of the case, as official returns show, are that far greater attention is paid to the requirements of the Margarine Act and Sale of Food and Drugs Act in this city than in any other urban or rural sanitary district in the country, special inspectors being appointed for the purpose; hence frauds are promptly dealt with and brought to light. Absence of prosecutions does not imply absence of fraud, as you naively infer, but rather that the Act is a dead letter. The official reports of the Local Government Board may lead you to modify the assertion that "Liverpool is ten times worse than London." If you will be good enough to insert this correction I shall be obliged. E. W. HOPE, M.D., Assistant Med. Officer.

Sanitary Department, Municipal Buildings, Dale Street, Liverpool, January 25th, 1893.

